# Nursing Students' Experiences With Artificial Intelligence: A Qualitative Study on Education, Clinical Practice, and Future Expectations

**DOI:** 10.1111/jep.70413

**Published:** 2026-03-15

**Authors:** Necibe Dagcan Sahin, Mehmet Yildirim

**Affiliations:** ^1^ Department of Nursing Fundamentals, Faculty of Health Sciences Kütahya Health Sciences University Kütahya Türkiye; ^2^ Department of Nursing, Faculty of Health Sciences Kütahya Health Sciences University Kütahya Türkiye

**Keywords:** artificial intelligence, clinical practice, nursing education, nursing students, qualitative research

## Abstract

**Background:**

With the rapid advancement of technology in healthcare, artificial intelligence (AI) has become an important tool in nursing education and clinical practice. However, there is limited knowledge regarding nursing students' experiences with AI, its areas of use, and their expectations.

**Aim:**

This study aimed to explore nursing students' experiences with AI and to reveal its role in nursing education, clinical practice, and future expectations.

**Methods:**

A qualitative phenomenological design was used. Face‐to‐face interviews were conducted with 14 nursing students from a state university who had been using AI between June and August 2025. Data were collected using a Demographic Information Form and a Semi‐Structured Interview Form. The interviews were recorded, transcribed verbatim, and analyzed using Braun and Clarke's thematic analysis method with the support of MAXQDA software.

**Results:**

Six main themes and fourteen subthemes emerged: (1) First encounters with AI and sources of information, (2) AI in nursing education, (3) AI in clinical practice, (4) Benefits of AI, (5) Limitations and concerns regarding AI, and (6) Future expectations and recommendations.

**Conclusion:**

Nursing students perceived AI as a supportive tool but emphasized that it cannot replace human‐centered care, empathy, or therapeutic communication. The findings highlight the need to integrate AI into nursing education and clinical practice within an ethical and pedagogical framework.

## 1 Introduction

Artificial intelligence (AI) has become a technology that has attracted considerable interest and discussion in higher education in recent years. While its roots date back to the 1950s, its impact in academia has become particularly evident in the last three decades [1]. AI's expressive capabilities in various text types and natural interactions have made it appealing to users [2]. AI, which has become widespread in many fields, especially the healthcare sector, aims to implement human‐specific perception and decision‐making processes through computer algorithms and machine learning [3].

The nursing profession is becoming increasingly complex due to the need to provide services across various specialties, patient diversity, and dynamic changes in the healthcare environment. Therefore, nurses are expected to be more equipped [4]. AI has led to significant changes in the fields of healthcare and nursing. Intelligent robots that alleviate nurses' workload improve the quality of care, while rehabilitation and social robots improve patients' physical capacity and support social interactions [5]. Advanced AI‐based models reduce manual data entry time, limit errors that may arise from nurses' subjective assessments, and improve the prediction of clinical outcomes by more accurately determining urgency levels [6]. Robotic technologies play a particularly active role in intensive care, rehabilitation, and patient management [7]. These technological advances are expected to lead to significant transformations in the field of nursing over the next decade [8]. However, limitations such as information security, ethical concerns, and the risk of misinformation should also be considered [7]. While AI systems are used effectively in diagnosis, education, and data security, their functions are currently limited to certain tasks [3].

AI contributes to student development through personalized learning and virtual applications in nursing education. Although AI improves the quality of care and simplifies processes in nursing, its impact on student processes in education and clinical settings has been limitedly studied [9]. A review of the literature reveals that nurses [5, 10, 11] and nursing students [12, 13] have generally positive attitudes toward AI. Previous research on AI use has focused largely on the perceptions of nurses and nursing students. However, studies comparing students' knowledge, attitudes, and experiences regarding AI integration in education and clinical practice are quite limited [14]. Global studies highlight a consistent gap between high AI interest and low formal knowledge among healthcare students. While nursing students in China, Pakistan, and Arab countries exhibit positive intentions, the majority report a significant lack of curricular exposure [5, 15, 16]. Similarly, research involving medical, dental, and veterinary students across Europe and Asia reveals that although they possess basic digital literacy, 76% have received no formal AI training, leaving them unprepared for AI‐related ethical dilemmas and clinical applications [17]. These findings underscore a universal need for integrating AI into health sciences curricula to bridge the gap between interest and professional competence. Therefore, this study is expected to offer a unique contribution to addressing existing limitations in the literature by examining nursing students' educational and clinical experiences related to AI from a multidimensional perspective. In this context, the study aims to help understand the opportunities and challenges experienced by students during the digital transformation of nursing education and to lay the groundwork for developing strategies for integrating AI into curricula. Furthermore, it is expected to contribute to the shaping of educational policies by providing data based on student experiences to train future nurses who will adapt to technological advancements.

The aim of this study is to examine nursing students' experiences with AI and to reveal the role of AI in nursing education, clinical practice, and future expectations.

## 2 Methods

The reporting of this study followed the Consolidated Criteria for Reporting Qualitative Research (COREQ) checklist, ensuring transparency across key areas such as the research team and reflexivity, study design, and the process of analysis and findings [18].

### 2.1 Setting and Sample

The study is grounded in a phenomenological epistemology, which focuses on describing the essential structures of human experience as they are directly lived. In this context, descriptive phenomenology was utilized to capture the ‘life‐world' of nursing students and to uncover the essential structures of their experiences with AI as they perceive them [19, 20, 21]. A qualitative design with a Husserlian descriptive phenomenological approach was employed in the study to describe the universal essence of nursing students' experiences with AI without prior interpretations or theoretical preconceptions [19]. It was conducted between June and August 2025 with first‐, second‐, third‐, and fourth‐year students aged 18 and above, enrolled in the Nursing Department of a university, who volunteered to participate. Additionally, only students who spoke Turkish and possessed knowledge about AI were included. The study group was determined using criterion sampling, one of the purposive sampling methods. Accordingly, diversity in age, gender, and types of AI experience was taken into account when selecting the sample [22, 23]. In qualitative research, sample size is determined at the point when data begin to repeat and no new information emerges‐that is, when data saturation is reached [24]. In this study, preliminary data saturation was observed during the 12th interview as no new themes or significant insights emerged. To ensure the comprehensiveness of the data, two additional interviews were conducted, and total data saturation was achieved after interviews with 14 nursing students, and the study was concluded. This sample size is consistent with the recommendations for descriptive phenomenological studies, which emphasize deep, rich descriptions from a relatively small, homogeneous group to capture the essence of a phenomenon rather than seeking statistical generalizability. Literature suggests that in phenomenological research, a sample size between 5 and 25 participants is often sufficient to reach thematic saturation [24, 25, 26].

### 2.2 Data Collection

Data were collected through in‐depth, one‐on‐one interviews using a Demographic Information Form and a Semi‐Structured Interview Form. The interviews were conducted in Turkish by the first researcher, who had prior experience in qualitative interviewing, and were audio‐recorded with a voice recorder. Each interview lasted approximately 20–40 min, and a total of 14 nursing students participated. Interviews were carried out in a quiet, well‐lit, and well‐ventilated private room, with only the researcher and the participant present. During the interviews, the researcher also took detailed observational notes in addition to recording the participants' verbal statements [24, 27].

#### 2.2.1 Demographic Information Form

The information form contains questions related to the participants' age, gender, grade point averages (GPA), the type of high school they graduated from, and their current class level.

#### 2.2.2 Semi‐Structured Interview Form

In order to thoroughly examine the participants' knowledge levels about AI, their experiences, and their thoughts on AI, fifteen open‐ended questions were prepared (Table 1). The interview guide was structured to cover the participants' technical background, clinical experiences, and future outlook. For instance, participants were asked about the AI tools they use most frequently and the sources of their AI knowledge. To delve deeper into the clinical dimension, questions were directed toward the role of AI in patient care and the specific challenges faced during its integration into clinical skills. Finally, the interviews explored students' future expectations and their recommendations for enhancing AI education within the nursing curriculum. Questions were formulated in a clear and accessible language, taking into account the comments of field experts and the findings from pilot interviews conducted with students. This allowed participants to engage in a comprehensive discussion about the role of AI in nursing education and clinical practice, the challenges they faced, and their expectations for the future [24, 27].

**Table 1:** Semi‐structured interview questions.

S1. Have you received any training on AI? From which sources did you obtain information?
S2. Which AI tools do you use most frequently?
S3. In which areas do you most often seek information through AI applications?
S4. What role do you think AI plays in the nursing profession?
S5. What expectations do you have in this regard?
S6. In which areas of nursing education do you use AI applications?
S7. In which areas of your theoretical education do you use AI applications?
S8. Do you have any experience in using AI technologies in clinical practice? If yes, what was this experience like?
S9. How has your knowledge of AI affected your clinical skills or patient care?
S10. Are there any challenges you have faced when integrating the knowledge you have gained about AI into the patient care process?
S11. What are the biggest challenges related to AI applications?
S12. Do you expect AI technologies to play a greater role in the nursing profession in the future?
S13. What are your expectations and concerns regarding this technology?
S14. In which areas do you think we need more knowledge or educational support regarding AI?
S15. What are your recommendations for more effective use of AI technologies in nursing education?

### 2.3 Data Analysis

Quantitative data were analyzed using SPSS version 25.0. Descriptive statistics, including frequencies, percentages, means, and standard deviations, were first calculated to comprehensively present the demographic characteristics and clinical education experiences of the participants.

Qualitative data analysis was conducted using a phenomenological thematic analysis approach, following the six‐step framework developed by Braun and Clarke (2006). This allowed the researchers to systematically identify themes while maintaining a focus on the essential descriptive structures of the participants' experiences. The transcriptions of the audio recordings were examined in detail, and preliminary codes were developed independently by two researchers. The data obtained after coding were categorized based on similarities, and main themes reflecting the study results were created. To ensure the reliability of the study, both researchers coded the transcripts and evaluated them until they reached consensus on the differences. Additionally, to strengthen the validity, the views of five experts were obtained. The identified themes were re‐evaluated and reported to accurately reflect the participants' perspectives. [28] Microsoft Office Word 2019 was used during the transcription, coding, and theme creation stages, and MAXQDA software was utilized in the data analysis process. In alignment with descriptive phenomenology, the researchers applied bracketing by keeping a reflexive journal and setting aside their personal beliefs about AI to allow the participants' authentic voices to emerge during the thematic analysis [19, 29]. Generative AI tools were used only for grammar and language editing purposes. No AI tools were used for content generation, data analysis, or interpretation. No generative AI tools were used to create or modify any figures, images, or artwork in this manuscript.

### 2.4 Rigor and Trustworthiness

In this study, the concepts of Reliability, Transferability, Dependability, and Confirmability identified by Lincoln and Guba (1989) were used. Multiple strategies were employed in the study to enhance the validity, reliability, transferability, and confirmability of the findings. Firstly, the interview transcripts and summaries of the final themes were shared with the participants, and their accuracy was verified through member checks. This step supported the reliability and robustness of the findings. To ensure consistency among the students, a standard questionnaire and audio recording device were used. All decisions made during data collection and analysis were systematically recorded, thereby contributing to the auditability of the research, and expert opinions were solicited to support the transparency of the research [20, 24, 27].

### 2.5 Research Team and Reflexivity

The study was conducted by a principal investigator who holds a doctorate degree and has extensive experience in qualitative research and student‐centered studies, along with other members skilled in qualitative methods. Considering that the professional experiences and prior experiences of the research team could influence the data collection and analysis processes, necessary precautions have been taken to minimize these effects.

### 2.6 Ethical Considerations

Ethical approval for the study was obtained from the Kütahya Health Sciences University Non‐Interventional Research Ethics Committee (Date: May 29, 2025; Decision Number: 2025/07‐26). Additionally, formal institutional permission was secured from the Kütahya Health Sciences University, Faculty of Health Sciences, Department of Nursing. Participants were informed in advance about the study's purpose and the use of audio recording devices, and both written and verbal informed consent were secured. To maintain confidentiality, each participant was anonymized using codes such as Student 1 (S1), S2, …, S14 during the interviews. The study was conducted in accordance with the ethical principles outlined in the Declaration of Helsinki.

## 3 Results

The study sample consisted of 14 students. Of the participants, 35.7% were female and 64.3% were male. The mean age was 22.64 ± 1.67 years, with ages ranging from 18 to 26. The mean cumulative GPA was 3.25 ± 0.19 (Table 2).

**Table 2:** Participants' descriptive characteristics (*n* = 14).

Descriptive characteristics	Category	*n*	%
Age (22.64 ± 1.67)	18–20	1	7.14
	21–23	9	64.29
	24–26	4	28.57
Gender	Female	5	35.7
	Male	9	64.3
GPA (3.25 ± 0.19)	2.51–3.00	1	7.14
	3.01–3.50	12	85.71
	3.51–4.00	1	7.14
Type of High School	Anatolian High School	8	57.14
	Science High School	2	14.29
	Health Vocational High School	4	28.57

**Figure 1:**
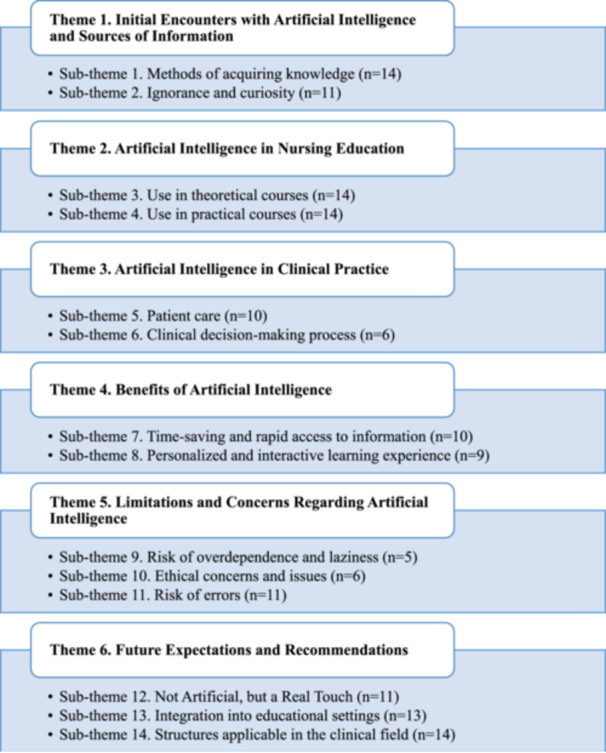
Themes and Sub‐Themes Related to Students' Perceptions and Attitudes Towards AI. *Note:* n indicates frequency.

Six main themes and their respective subthemes related to nursing students' perceptions and experiences of AI were identified in this study (Figure 1).

### 3.1 Theme 1. Initial Encounters With AI and Sources of Information

This theme encompasses students' first experiences with AI, their processes of obtaining information about AI, and how they utilize AI as a source of knowledge.

#### 3.1.1 Sub‐Theme 1. Methods of Acquiring Knowledge

Students reported that they had not received formal training on AI; however, they possessed a general awareness of the subject through information acquired from the internet and their social circles.

I have not received any formal training on AI. I gained knowledge through the internet and my friends.

S3, 4th year, female

I have not received any formal training on AI; however, due to the influence of my social environment, I have gained some knowledge on this topic. In addition, thanks to the internet—which we all use frequently—it is possible to access AI‐related news and applications through certain platforms.

S4, 3rd year, female

Since I have been involved with computers since childhood, I was able to grasp the logic of AI on my own. I only obtained information about its use from YouTube. Apart from that, I think its use is quite simple.

S14, 4th year, male

I have not received any training. Like everyone else, I started using it later in life—perhaps for the past 6 years—and since then, I have been using it much like a Google search engine.

S13, 4th year, male

#### 3.1.2 Sub‐Theme 2. Ignorance and Curiosity

Students expressed high levels of curiosity toward AI and stated that they needed more information in this field.

We need more information about AI applications, how they work, and how we can use them. I do not fully know how to use it, and I am curious.

S5, 3rd year, female

I am curious and I enjoy learning through personal experimentation. I want to gain knowledge about new tools and technologies.

S14, 4th year, male

### 3.2 Theme 2. AI in Nursing Education

This theme highlights the role of AI in bridging what students learn in their education with the applications they encounter in clinical practice.

#### 3.2.1 Sub‐Theme 3. Use in Theoretical Courses

Students reported using AI in theoretical courses to access information quickly and efficiently. They noted that AI applications facilitated their learning process, particularly in accessing academic resources, researching concepts, and preparing presentations.

I use AI to access resources related to my theoretical education. Sometimes, the necessary resources exceed my budget, so AI helps me access such content. Overall, I prefer using it to make my information‐gathering process easier.

S3, 4th year, female

I use AI to research topics and terms I do not understand in nursing education. It also facilitates my learning process as it can present topics through concept maps or visual representations.

S4, 3rd year, female

For example, I have a 500‐page exam note that I need to summarize. I ask AI to summarize it, and it turns the 500 pages into approximately a 10‐page condensed material. This gives me significant flexibility in my study process.

S6, 4th year, male

AI helps me a lot when preparing presentations. For instance, when I specify the topic for the presentation, AI organizes the content very systematically. All that remains for me is to design it, and there are separate applications available for that as well.

S8, 4th year, male

#### 3.2.2 Sub‐Theme 4. Use in Practical Courses

Students indicated that they used AI in practical courses to obtain support on disease symptoms, medication information, and special cases; to prepare care plans; and to write reports.

I ask questions about patient care. When preparing a care plan, I use AI to learn about nursing interventions relevant to the diagnosis I have identified. I also request examples when writing reports or preparing written assignments, which helps me complete my assignments more easily and quickly.

S3, 4th year, female

I also occasionally seek support regarding medications. Especially when AI creates tables containing information such as side effects and active ingredients, it helps me understand the essential and important points.

S2, 4th year, female

I think I have shortcomings especially in health and hospital management topics. I do not fully know what certain laboratory values mean. Our instructors sometimes ask us to fill out forms, and this is exactly where AI comes to my rescue.

S9, 2nd year, male

### 3.3 Theme 3. AI in Clinical Practice

This theme addresses students' perceptions of the impact of AI on patient care and clinical decision‐making processes in clinical settings.

#### 3.3.1 Sub‐Theme 5. Patient Care

Students stated that AI helped them correct misconceptions, enabling them to make more accurate decisions in patient care and thereby improving the quality of care.

First of all, if there is something I know incorrectly, AI can point it out to me. This allows me to make more accurate decisions in patient care and treatment practices. In this way, I aim to provide the best possible care without causing harm to the patient. Thanks to AI, I can correct my misconceptions and contribute to improving the quality of care, facilitating the patient's discharge under better conditions and in a shorter time.

S11, 3rd year, male

With the information I have gained through AI, I can now inform patients about their treatments. I not only provide information about the treatment itself but also explain which non‐pharmacological methods they can apply and how to apply them. I can now approach patient care from a more comprehensive and holistic perspective.

S12, 3rd year, female

#### 3.3.2 Sub‐Theme 6. Clinical Decision‐Making Process

Students expressed that AI applications supported them in developing solutions and facilitated decision‐making in clinical practice.

I use AI in the clinical decision‐making process. I see it as an application that helps me develop solutions and makes my decisions easier.

S3, 4th year, female

### 3.4 Theme 4. Benefits of AI

This theme reflects students' perceptions of the benefits and contributions of AI.

#### 3.4.1 Sub‐Theme 7. Time‐Saving and Rapid Access to Information

Students stated that AI saves time and enables quick and easy access to information.

I think AI is beneficial because it saves time and provides easy access to information.

S3, 4th year, female

In the past, books were used to obtain information, then the internet and search engines became widespread. Today, AI is being used more and more. With the technological advancements over the years, AI has become a highly practical tool.

S6, 4th year, male

AI offers practical, fast, and easy use. It makes accessing information very convenient. It can prepare resources quickly and in an organized manner on my behalf, and because it has a better command of grammar than I do, I enjoy using it.

S8, 4th year, male

#### 3.4.2 Sub‐Theme 8. Personalized and Interactive Learning Experience

Students noted that interacting with AI made them feel as if they were conversing with a real person.

When I ask something on Google, it feels like I am conducting a general, publicly accessible search. But when I ask ChatGPT, I feel as though there is someone in front of me and I am having a one‐on‐one conversation. This personal and sincere feeling makes communicating with AI a more comfortable and pleasant experience for me.

S1, 4th year, male

AI is a system that allows you to ask questions and gives the impression that someone is listening to you. Its constant attentiveness and effort to respond to every question can make it feel almost like a friend. This, in turn, helps me learn more

S9, 2nd year, male

Just like we ask a friend a question, we can ask AI and receive answers or suggestions in return. Sometimes, it can even articulate our own thoughts. Overall, AI acts both as a friend and as an educational guide, which also makes it a highly effective tool for language use.

S11, 3rd year, male

### 3.5 Theme 5. Limitations and Concerns Regarding AI

This theme encompasses the limitations of AI as identified by students, including the risks of overdependence and professional complacency, ethical issues, and the potential for errors.

#### 3.5.1 Sub‐Theme 9. Risk of Overdependence and Laziness

Students expressed concern that excessive reliance on AI could diminish professional responsibility and lead to occupational complacency.

I believe that AI can have generally negative or worrying effects in the field of nursing. If it is used very frequently and intensively, risks such as dependency may arise. For example, if nursing students begin to place too much trust in AI, they may gradually relegate their own responsibilities to the background. This could lead to negligence of duties, laziness, and irresponsibility in clinical settings. The tendency to delegate everything to AI may result in a decline in professional attentiveness and diligence.

S11, 3rd year, male

The risk of AI causing individuals to become unsocial and lazy by distancing them from social interaction cannot be ignored.

S3, 4th year, female

#### 3.5.2 Sub‐Theme 10. Ethical Concerns and Issues

Students noted that AI raises uncertainties regarding privacy and data security, creating potential risks in protecting personal information.

The issue of protecting personal information inevitably raises concerns. For example, when we integrate AI into some of our applications, questions arise such as, ‘Could my other conversations be seen by others?' or ‘Could this data be transferred to other platforms?' Such situations naturally create a sense of fear and distrust regarding data security.

S1, 4th year, male

Although I cannot be certain of the accuracy, when we start using AI we often encounter ‘cookie' notifications. While this may appear to be a routine feature of an application, it could nonetheless lead to ethical problems.

S9, 2nd year, male

One concerning aspect of AI is the potential rise in cybercrime and the ease with which unauthorized individuals could access personal data. This can cause serious problems from both ethical and security perspectives. Ultimately, the lack of trust and the absence of a clear framework for how humanity will manage this technology increase the concerns surrounding AI.

S3, 4th year, female

#### 3.5.3 Sub‐Theme 11. Risk of Errors

Students observed that AI can sometimes provide inaccurate or incomplete information, emphasizing the need for careful verification of AI‐generated content.

Sometimes AI can give incorrect information. Therefore, I cannot simply copy and paste the text it produces without reading it. Information that initially appears correct can later shift in a completely different direction, and I may realize it is wrong. For this reason, I always make sure to review and check it.

S2, 4th year, female

If the information provided by AI contains errors or omissions, it can complicate our work rather than simplify it. This is because the healthcare field often does not tolerate mistakes and requires great attention to detail.

S3, 4th year, female

### 3.6 Theme 6. Future Expectations and Recommendations

This theme reflects students' suggestions regarding the integration of increasingly prevalent AI applications into the nursing profession, education, and clinical settings.

#### 3.6.1 Sub‐Theme 12. Not Artificial, but a Real Touch

Students emphasized that genuine patient communication, empathy, and emotional connection cannot be achieved through AI. They highlighted the significance of therapeutic touch and human interactions, such as eye contact, in patient care.

Therapeutic touch—approaching the patient gently, establishing eye contact, and communicating through touch—leaves a positive impression on the patient and contributes positively to the treatment process. Can AI do this?

S7, 4th year, male

For example, when we ask a patient ‘How are you? Are you feeling well?', we can see how happy they become and how much this question helps. I do not think AI can provide this type of emotional interaction. Therefore, I do not believe it can replace nurses or the nursing profession.

S1, 4th year, male

Empathy is essential; one must understand the patient in that moment and establish a connection. Since AI currently has no emotions, it is not capable of building such a bond.

S6, 4th year, male

#### 3.6.2 Sub‐Theme 13. Integration Into Educational Settings

It was suggested that AI should be more widely introduced, that both students and nurses should receive training on its use, and that specialized AI engines based on reliable sources should be developed.

It is important to promote AI more broadly, provide training on the subject, further develop the technology to deliver more accurate and complete information, and raise awareness among students and nurses so they can use this technology effectively.

S3, 4th year, female

In my opinion, in nursing education, AI could be used by educators in case presentations, enabling students to learn cases in a more memorable way. Moreover, using AI in practical nursing courses and laboratory sessions could facilitate easier understanding of the material and allow all students to practice, thus saving time.

S3, 4th year, female

For AI to be used effectively in nursing education, developing a specialized AI engine focused on nursing would be highly beneficial.

S9, 2nd year, male

#### 3.6.3 Sub‐Theme 14. Structures Applicable in the Clinical Field

Students noted that AI could support nurses with drug information and in challenging disease scenarios.

I believe AI could provide more assistance in clinical settings by offering information about medications and guiding us on how to act when faced with challenging or unfamiliar diseases and conditions.

S4, 3rd year, female

AI could be utilized particularly in preparing care plans, identifying diagnoses, and in intervention and evaluation stages.

S9, 2nd year, male

For example, AI could assist in interpreting laboratory findings, analyzing X‐ray results, and preparing care plans.

S10, 3rd year, male

Given its broad scope, AI could even create a virtual hospital and laboratory environment for us. In this virtual space, we could apply our knowledge, recognize our mistakes, and gain valuable experiences, which we could later apply in real‐life practice. AI offers us a significant opportunity in this regard.

S11, 3rd year, male

AI technologies could also be developed for patient use, creating patient‐support systems they can benefit from. Due to workload, nurses may sometimes be unable to provide patients with sufficient information. Therefore, a specialized AI system that delivers daily information to patients about their illnesses and treatments would be beneficial.

S12, 3rd year, female

## 4 Discussion

According to the study findings, students stated that they had not received training in AI, but they had a general awareness of AI thanks to information they acquired online and from their surroundings. Students also stated that they needed to learn more about AI. As technology increasingly becomes central to healthcare today, it is crucial for nursing students to be equipped with strong digital knowledge and skills to adapt to this digital transformation [30]. In a study, it was stated that approximately half of the nurses had knowledge about AI; this knowledge was mostly acquired through self‐learning, various courses, and postgraduate courses [31]. In contrast to our study findings, a meta‐analysis reported that most students had limited knowledge of AI [32]. Similarly, a study conducted with students and healthcare professionals found that a large portion of participants had low knowledge of AI [5]. These differing results may be due to the fact that AI is a relatively new field in health education, that different studies included diverse sample groups, and that students' experience and knowledge levels regarding AI vary.

In the study, students reported using AI to access information quickly and practically in theoretical courses, to obtain information about disease symptoms, medication information, and special situations in practical courses, and to prepare care plans and write reports. Studies have also examined nursing students' opinions on using AI in their education [9]. Similar to our study findings, a study conducted with nursing students also indicated that students used AI to access accurate information, clarify nursing concepts, and obtain information on topics such as diagnosis and health assessments [33]. Additionally, studies indicate that nursing students are interested in AI, but this interest is not sufficiently reflected in their educational programs. It is emphasized that AI is not fully integrated into the curriculum, and digital competencies are not standardized. Low levels of digital literacy stand out as a factor limiting the effective use of technology [30]. Findings and studies suggest that the proper integration of AI has the potential to transform nursing practice and advance patient care [34]. AI and chatbots have a powerful power to transform nursing education and the nursing profession. Educators must understand the opportunities these technologies offer and integrate them into their educational processes [3]. The findings reveal that nurses need a strong knowledge base to adapt to these developments [35].

According to the findings of the study, students stated that AI improves the quality of care. They also stated that AI applications develop solutions in the clinical decision‐making process and facilitate decision‐making. A review of the literature revealed no studies examining students' AI experiences in clinical practice. These studies primarily focus on nurses. One study reported that AI reduces the time lost due to nurses' administrative duties, improving efficiency and patient outcomes in areas such as intensive care and emergency rooms [10]. Furthermore, it has been reported that nurses can focus more on care processes through the use of AI, and administrative burden is reduced through automation [36]. AI holds significant potential for improving the quality of care and improving clinical processes by making work more efficient in the healthcare field [37]. While these findings point to the positive impact of AI on nursing care, they also indicate that nursing students' experiences with AI in clinical practice have not yet been adequately researched. This highlights the need for further investigation of student‐focused AI applications and experiences.

It is reported that AI, with its ability to quickly analyze and access data, allows students to focus on more critical aspects of research [38]. This study determined that AI saves time and allows students to access information more quickly. Furthermore, the literature indicates that AI speeds up processes in the healthcare field, reducing costs while enabling more efficient use of energy and time [37]. This study also observed that students interacting with AI applications enhanced their learning processes, as if they were working with a friend. A study conducted with nursing students reported that students initially struggled to use AI due to a lack of knowledge and experienced emotional turmoil. However, over time, students began to use AI as a supportive tool in shaping their ideas, their research interests increased, and they developed scientific thinking [39]. These findings show that AI plays an important role both in accelerating learning processes and in gaining value as a supportive tool for students. Viewing AI as a ‘friend' suggests that students value a safe, non‐judgmental feedback loop that reduces learning anxiety in high‐stakes clinical education. Students particularly appreciate AI for providing ‘low‐stakes' assistance and a sense of convenience during independent study [40]. However, literature indicates a more nuanced perception; students remain cautious due to concerns regarding accuracy, misinformation, and the potential loss of foundational nursing competencies [41, 42]. Consequently, this ‘friendship' is limited by a lack of absolute trust and a perceived need for human oversight to manage complex ethical dilemmas and maintain academic integrity [40, 41].

The study's emphasis on the risks of addiction and laziness among students towards AI is quite striking. Similar to our study findings, it has been reported that students' excessive reliance on AI tools like ChatGPT can impede the development of critical thinking, problem‐solving, and innovation skills, weakening their independent thinking abilities [2]. It has also been noted that excessive reliance on AI can lead to a decline in core competencies such as critical thinking and problem‐solving in healthcare professionals, and can lead to laziness [37]. Educators also note that students' excessive reliance on AI can lead them to adopt a passive approach rather than active learning, weakening their analytical thinking and practical problem‐solving abilities [43]. These findings highlight that, despite the advantages AI offers in education, excessive use can lead to a decline in students' critical thinking and problem‐solving skills. Students' fear of 'laziness' suggests they perceive AI as a cognitive shortcut that threatens clinical reasoning. Literature supports this, noting that mechanical reliance on AI outputs can lead to a decline in independent analysis and risks to academic integrity [44]. To prevent this, AI should be implemented as a supplementary tool (augmented intelligence) that supports, rather than replaces, human judgment [45].

In nursing education, there is a strong emphasis on the application of fundamental ethical principles such as patient privacy, professional obligations, and professional ethical values [41]. In this study, students reported uncertainties regarding AI data privacy and security, risks to the protection of personal information, and concerns about cyberattacks. A review of findings also reveals that healthcare students have ethical concerns, particularly regarding patient privacy and data management and storage, in clinical AI applications [14]. Patient privacy and the protection of ethical principles are particularly emphasized in clinical practice [41]. A review of the literature indicates that a lack of conceptual clarity regarding AI creates various challenges in both educational and practical applications, limiting the safe and ethical use of technology [46]. Although AI‐supported chatbots offer certain advantages, they may also lead to trust issues [47]. Considering that AI in healthcare carries risks such as error‐prone algorithms, data bias, lack of transparency, and ethical concerns [37], it is suggested—based on the research findings and existing literature—that patient confidentiality, data privacy, and ethical principles should be prioritized in educational processes to ensure the safe, ethical, and transparent use of AI.

In the study, students highlighted the risk of AI providing inaccurate or incomplete information. Literature suggesting that overreliance on AI can negatively impact patient trust [48] confirms students' concerns. [9] This situation aligns with the uncertainties and ethical concerns surrounding the reliability and accuracy of AI algorithms in the literatüre [46]. New AI‐based tools are still limited in terms of the number and quality of resources they provide and sometimes carry the risk of inaccurate citations [41]. Furthermore, the need for error‐free and accurate data use in healthcare aligns with studies arguing that AI‐based clinical decision support systems can only operate reliably under human supervision [49]. The increasing influence of AI in clinical processes may lead to an overreliance on technological solutions rather than critical thinking in nursing practice. The need for students to verify information from AI supports the need to balance technology with human judgment [9]. These findings reveal that, in addition to the advantages offered by AI technologies in clinical applications, caution should be exercised regarding accuracy and reliability.

Studies show that AI technologies can support the patient care process by assuming some tasks in nursing, but they cannot provide nurses with human‐centered care elements such as empathy, emotional connection, and therapeutic touch [50]. In this study, students emphasized that AI cannot replace nurses. These views align with Watson's nursing theory and the concept of human‐centered care [9, 51]. Another study states that AI can transform nursing and improve the quality of care, but cannot replace nurses' human‐centered care and advocacy roles [37]. Similar results are evident in the literatüre [52]. In contrast, a study examining the impact of mental health on the care process concluded that negative emotions experienced by nurses, such as stress and hopelessness, affect the quality of care; therefore, AI can make positive contributions to care if it conveys emotional content [47]. These findings suggest that AI can offer significant support in nursing practice but cannot replace the fundamental elements of human‐centered care. These results suggest that AI does not threaten the nursing profession but rather redefines it, allowing nurses to reclaim their core ‘human’ identity by automating repetitive tasks. Literature supports this shift, arguing that integrating AI allows for a reinvestment in humanistic practice where technology handles data while nurses focus on emotional care [53]. Furthermore, by moving from passive users to active co‐creators of ethical AI, nurses can ensure that technology amplifies, rather than replaces, the ‘human heart’ of healthcare [54, 55].

The study's recommendation to integrate AI into nursing education through case studies, simulations, and theoretical courses aligns with the literature's recommendation that AI literacy modules and practical training should be included in curricula [46, 56, 57]. Furthermore, another study suggests integrating AI into education through policy development and inter‐institutional collaboration [30]. Furthermore, the need to develop specialized nursing‐focused AI engines and verify their accuracy with reliable sources is important from both practical and ethical perspectives [41]. This highlights the importance of providing professional development support for educators to effectively use technology. In addition, it is of great importance to create environments where nurses can effectively use and evaluate AI models and to integrate AI education into the nursing curriculum with state‐supported policies [6, 56].

The findings of studies suggesting that AI can support students in areas such as drug information, patient monitoring, and laboratory result interpretation in clinical settings are supported by studies in the literature suggesting that AI reduces workload in routine clinical tasks and improves patient care when used as decision support systems [49, 57]. Furthermore, the recommendation to create virtual hospital and laboratory environments reflects the increasing importance of AI‐assisted simulation and virtual reality applications in health education after the pandemic [58]. One study demonstrates the potential of AI‐assisted triage to increase accuracy and support nurses' decision‐making processes. Although AI cannot replace human interaction, it can significantly improve predictive power [6].

### 4.1 Strengths and Limitations

One of the study's most significant strengths is its in‐depth, qualitative examination of nursing students' AI experiences. The inclusion of participants of varying ages, genders, and AI experiences through a purposive sampling method increased the diversity and scope of the findings. Furthermore, the use of a semi‐structured interview form during data collection allowed participants to express their opinions freely and in detail. However, the study has several limitations. The limited sample size limits the generalizability of the findings. Furthermore, the inclusion of students from only one university made it difficult to reflect experiences across different educational settings. The scope of the interview questions may have limited the ability to obtain more in‐depth information on the specific impact of AI on clinical practice in some cases. Future research is recommended to collect data with larger and more diverse sample groups.

## 5 Conclusion

The research presents a comprehensive account of nursing students' experiences and views on AI technologies. Students have indicated that AI offers quick access to information, time management, and personalized learning opportunities in educational and clinical settings. On the other hand, the limitations of artificial intelligence, such as the risk of errors, ethical issues, and over‐dependence, have also been discussed. Students concluded that while they are using AI in their courses and practices, it cannot replace the real human touch and therapeutic communication in nursing. It has been emphasized that there is a need to increase information and training support for more effective use of AI technology in clinical and educational environments. Although the opportunities provided by AI in nursing practices are evident, findings suggest that ethical and practical limitations should not be overlooked. In light of the results, the importance of developing strategies by educators and healthcare professionals to strengthen the integration of AI in nursing practices has been emphasized. Specifically, concrete strategies such as integrating AI ethics into the curriculum, utilizing AI‐driven simulations, and establishing a digital nursing competency framework are recommended to ensure safe and effective AI integration in nursing education.

## Author Contributions

**Necibe Dagcan Sahin:** conceptualization, methodology, data curation, investigation, visualization, writing – original draft preparation, writing – reviewing and editing, supervision, validation. **Mehmet Yıldırım:** Conceptualization, methodology, data curation, investigation, visualization, writing – original draft preparation, writing – reviewing and editing, validation.

## Funding

The authors received no specific funding for this work.

## Consent

Institutional permission for the study and the subsequent publication of its findings was obtained from the Kütahya Health Sciences University, Faculty of Health Sciences. All participants were informed that the data obtained from the interviews would be used for scientific publication purposes in an anonymized format. Written and verbal informed consent, including the permission to publish de‐identified quotations, was obtained from all individual participants included in the study. All participants provided both written and verbal informed consent prior to their inclusion in the study.

## Conflicts of Interest

The authors declare no conflicts of interest.

## Data Availability

Due to ethical and legal considerations, the dataset generated and analyzed during the current study is not publicly available. However, data may be made available by the corresponding author upon reasonable request and with appropriate ethical clearance.
